# Potential interaction between exogenous anabolic steroids and sugammadex: failed reversal of rocuronium in a patient taking testosterone and trestolone acetate

**DOI:** 10.1002/anr3.12262

**Published:** 2023-11-27

**Authors:** K. Farkas, A.‐C. Aeberhard, E. Schiffer, S. J. Brull, C. Czarnetzki, J. Maillard

**Affiliations:** ^1^ Department of Anaesthesiology, Pharmacology, Intensive Care and Emergency Medicine University Hospitals of Geneva Geneva Switzerland; ^2^ Department of Anaesthesiology, Pharmacology, Intensive Care and Emergency Medicine University Hospitals of Geneva Geneva Switzerland; ^3^ Department of Anaesthesiology, Pharmacology, Intensive Care and Emergency Medicine University Hospitals of Geneva Geneva Switzerland; ^4^ Department of Anaesthesiology and Perioperative Medicine Mayo Clinic College of Medicine and Science Jacksonville Florida United States; ^5^ Department of Anaesthesiology, Pharmacology, Intensive Care and Emergency Medicine University Hospitals of Geneva Geneva Switzerland; ^6^ Department of Anaesthesiology, Pharmacology, Intensive Care and Emergency Medicine University Hospitals of Geneva Geneva Switzerland

**Keywords:** neuromuscular nondepolarizing agents, steroids, sugammadex

**Figure 1:**
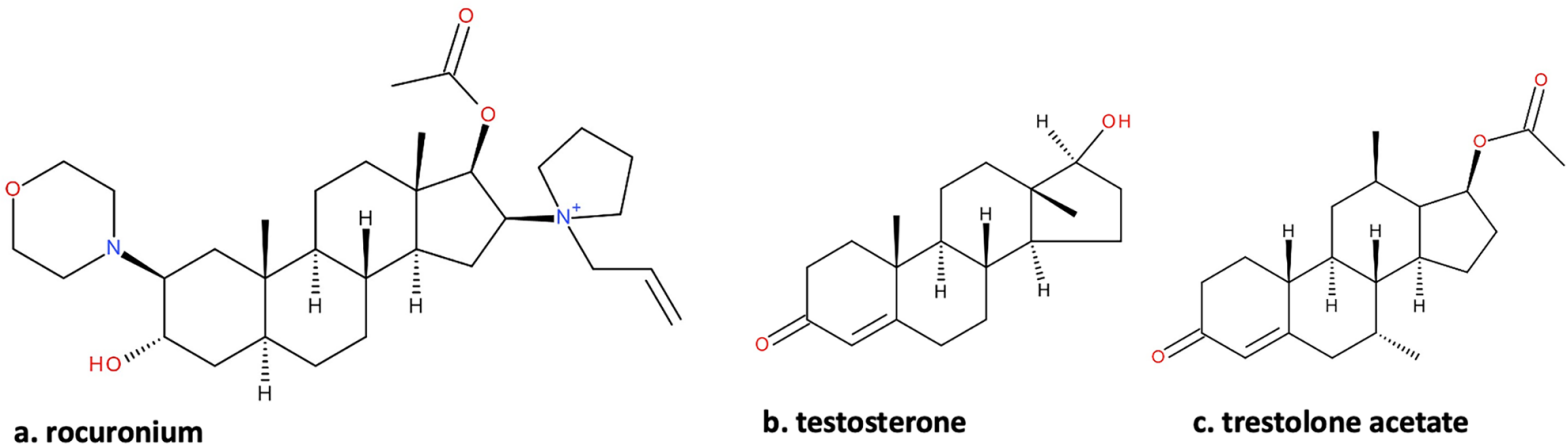
Comparison of chemical structures of (a) rocuronium, (b) testosterone and (c) trestolone acetate.

Sugammadex is a selective neuromuscular blocking agent (NMBA) binding drug which reverses neuromuscular block induced by aminosteroid non‐depolarising NMBAs. It contains a gamma‐cyclodextrin structure with a hydrophilic internal cavity into which aminosteroid NMBAs are bound with high affinity, thereby inactivating them (Fig. 1) [1]. However, sugammadex can also bind to other molecules [2]. Here, we report a failure of sugammadex antagonism of neuromuscular block with rocuronium in a patient who was taking exogenous steroid hormones.

A 60‐year‐old man, scheduled for a robot‐assisted nephrectomy, disclosed an ongoing use of steroids related to his bodybuilding practice. Self‐medication included testosterone (750–1000 mg per week intramuscularly) and trestolone acetate (300 mg per week intramuscularly). Trestolone acetate is a selective androgen receptor modulator and a nandrolone derivative, 10 times more potent than testosterone (Fig. 1). Preoperative testing revealed a free testosterone blood level of 5540 pmol.l^−1^ (reference value, 170–660 pmol.l^−1^) and total testosterone (sex hormone binding globulin, SHBG) of 134 nmol.l^−1^ (reference value, 6.1–27.1 nmol.l^−1^). The patient weighed 102 kg and was 180 cm tall, with normal renal function.

Routine general anaesthesia was provided for the robotic surgery, with a total rocuronium dose of 139 mg intravenously (60 mg at induction followed by infusion of 0.2 mg.kg^−1^.h^−1^). The baseline train‐of‐four ratio (TOFr) measured with acceleromyography (Philips IntelliVue NMT, Philips, Amsterdam, The Netherlands) before rocuronium administration was 100%. At the end of surgery, TOFr was 33%, requiring administration of 2 mg.kg^−1^ sugammadex. Ten minutes after administration of sugammadex 200 mg intravenously the TOFr had increased to 48%. After five more minutes, TOFr reached 52%. Due to this unusually slow reversal, an interaction between sugammadex and steroid hormones was suspected, and we supplemented the neuromuscular block antagonism with intravenous neostigmine 2.5 mg and glycopyrrolate 0.5 mg. Within 45 seconds of neostigmine administration, TOFr recovered to 100%.

This case describes what might be a faster‐than‐expected antagonistic effect of neostigmine; however, the onset of action of neostigmine administered at a recovery TOFr of 21% can be as quick as 40 sec [3]. This was consistent with our observations, particularly since neostigmine was given after sugammadex‐induced recovery had already started. Our case suggests the potential for pharmacological interactions that may reduce the efficacy of sugammadex in antagonising aminosteroid NMBAs. Anabolic steroids, such as testosterone or trestolone acetate, used to increase muscle mass, are increasingly popular. It is possible that these drugs or their metabolites, which share some of the structural properties of aminosteroid NMBAs, may bind to sugammadex thereby reducing its effectiveness.

Sugammadex is known to interact with endogenous steroid hormones: it reduces progesterone levels, prompting the advice for patients who take the oral contraceptive pill to use additional non‐hormonal contraception after administration [4], and one study reported a transient increase in endogenous testosterone after sugammadex administration [5]. However, there are no studies investigating this interaction between anabolic steroids and sugammadex, therefore our observations remain speculative. Other reasons for delayed reversal with sugammadex should be considered, as should neuromuscular monitor malfunction or erroneous readings. The potential effects of trestolone acetate, and in particular, its potential effects on the efficacy of sugammadex, should be studied further.
